# Spray-dried immobilized lipase from *Geobacillus* sp. strain ARM in sago

**DOI:** 10.7717/peerj.6880

**Published:** 2019-05-31

**Authors:** Nur Syazwani Mohtar, Mohd Basyaruddin Abdul Rahman, Shuhaimi Mustafa, Mohd Shukuri Mohamad Ali, Raja Noor Zaliha Raja Abd. Rahman

**Affiliations:** 1 Halal Products Research Institute, Universiti Putra Malaysia, Serdang, Selangor, Malaysia; 2 Enzyme & Microbial Technology Research Centre (EMTech), Universiti Putra Malaysia, Serdang, Selangor, Malaysia; 3 Department of Chemistry, Faculty of Science, Universiti Putra Malaysia, Serdang, Selangor, Malaysia; 4 Department of Microbiology, Faculty of Biotechnology and Biomolecular Sciences, Universiti Putra Malaysia, Serdang, Selangor, Malaysia; 5 Department of Biochemistry, Faculty of Biotechnology and Biomolecular Sciences, Universiti Putra Malaysia, Serdang, Selangor, Malaysia

**Keywords:** Sago, Lipase, Entrapment, Spray-dry, Enzyme immobilization, *Geobacillus* sp.

## Abstract

Sago starch is traditionally used as food especially in Southeast Asia. Generally, sago is safe for consumption, biodegradable, easily available and inexpensive. Therefore, this research was done to expand the potential of sago by using it as a support for enzyme immobilization. In this study, ARM lipase, which was isolated from *Geobacillus* sp. strain ARM, was overexpressed in *Escherichia coli* system and then purified using affinity chromatography. The specific activity of the pure enzyme was 650 U/mg, increased 7 folds from the cell lysate. The purified enzyme was immobilized in gelatinized sago and spray-dried by entrapment technique in order to enhance the enzyme operational stability for handling at high temperature and also for storage. The morphology of the gelatinized sago and immobilized enzyme was studied by scanning electron microscopy. The results showed that the spray-dried gelatinized sago was shrunken and became irregular in structure as compared to untreated sago powder. The surface areas and porosities of spray-dried gelatinized sago with and without the enzyme were analyzed using BET and BJH method and have shown an increase in surface area and decrease in pore size. The immobilized ARM lipase showed good performance at 60–80  °C, with a half-life of 4 h and in a pH range 6–9. The immobilized enzyme could be stored at 10 °C with the half-life for 9 months. Collectively, the spray-dried immobilized lipase shows promising capability for industrial uses, especially in food processing.

## Introduction

Sago is a starch extracted from the pith of *Metroxylon sagu* that is commonly found in the Pacific regions of Asia. It is traditionally used as a thickener and binder in the food and pharmaceutical industry and more recently has been increasingly utilized in the production of biodegradable polymers (Juna & Huber, 2012). The useful properties of sago starch are: (1) it is easy to gelatinize, as its gelatinization temperature is low; (2) it has a high viscosity; (3) it is easily molded; and (4) gel syneresis is low (Khatijah & Patimah, 1995; Ahmad et al., 1999). Sago is a type of natural polymer that can form a hydrogel when gelatinized. When heated in the presence of water, sago undergoes an irreversible order–disorder transition termed gelatinization. The granules swell, absorb water, lose crystallinity and leach amylose (Jenkins & Donald, 1998). Hydrogels are readily used as the supporting materials for immobilization or separation of proteins (Ha et al., 2012). Hydrogels are crosslinked polymer networks that can absorb a huge amount of water (Basri et al., 1999; Basri et al., 2001). The key parameters that allow hydrogels to be used as carriers for immobilized enzymes are the type and origin of the hydrogel, molecular structure and the physicochemical properties. Chitosan, alginate and gelatin are the most commonly used hydrogels for enzyme and microbial cell immobilization. These polymers are inexpensive, derived from natural sources, inert, strongly hydrophilic and biocompatible (Trusek-Holownia & Noworyta, 2015).

Immobilized enzymes are now favored for industrial use, as they have more advantages than the free enzymes in terms of storage stability, reusability, easy enzyme separation from products and reactants, low cost, fast termination of reactions and controlled products formation. In ester synthesis and transesterification, immobilized lipases also provide the essential non-aqueous conditions for the reactions (Akoh & Min, 2002; Christensen et al., 2003; Minteer, 2011). The techniques of immobilization frequently used include adsorption, covalent binding, encapsulation, entrapment and cross-linking (Christensen et al., 2003; Minteer, 2011; Adlercreutz, 2013; Brena, González-Pombo & Batista-Viera, 2013; Datta, Christena & Rajaram, 2013). Other than the conventional methods for immobilization, a recent method that is called layer-by-layer (LbL) self-assembly of nanofilms is gaining scientific interest. This method is generally to coat substrates with functional thin films that were assembled either by electrostatic interactions or molecular interactions (e.g., covalent, hydrogen-bonding, host-guest) with various materials like polymers, proteins, lipids and nucleic acids used as film elements (Richardson, Björnmalm & Caruso, 2015). The criteria for good support material for enzyme immobilization are based on having the mechanical strength, high loading capacity, cheap, chemically durable, allow the proteins to function and having a hydrophobic or hydrophilic character (Akoh & Min, 2002; Christensen et al., 2003; Minteer, 2011). When sago was used as the support for enzyme immobilization, the entrapment technique would be the best option rather than adsorption, covalent binding and cross-linking. This is because the sago is a non-ionic polysaccharide, and thus does not interact with charged compounds like the enzymes. Some of the techniques to entrap enzymes are gel or fiber entrapping and micro-encapsulation. Lipase has been immobilized via entrapment or encapsulation in *κ*-carrageenan and polysaccharide hydrogel beads in previous studies (Jegannathan, Seng & Ravindra, 2009; Kareem, Adio & Osho, 2014). A recent technique to encapsulate lipase is by synthesizing a protein-inorganic hybrid nanoflower system, in which the protein molecules form complexes with copper ions (Ge, Lei & Zare, 2012; Jiang et al., 2018; Kumar et al., 2018). Enzyme immobilization by entrapment within polymeric network enables substrates and products to pass through while trapping the enzymes, thus allowing substrate-enzyme contact to be achieved (Talekar & Chavare, 2012; Brena, González-Pombo & Batista-Viera, 2013). Using sago as the support for enzyme immobilization would have many benefits as it is safe for consumption, biodegradable, abundant in availability and cheap (Chin, Pang & Lim, 2012).

As proteins are more stable and can be stored for a longer time in solid form, freeze-drying or lyophilization have been adopted by researchers as they are perceived to be a gentle process for concentrating or drying biologically active substances (Roy & Gupta, 2004). However, spray-drying can be considered a superior alternative to freeze-drying when dehydrating a large quantity of samples as the process would take less time and be more economical (Alloue et al., 2007). Spray-drying has been widely used as a method to get fine or aggregated dry particles or to encapsulate active molecules from a liquid suspension. The quick thermal exposure during the aerosol process makes it suitable for the use of fragile organic moieties while the rapid drying allows the materials to stay in metastable states. One of the recent methods for producing high-performance heterogeneous catalysts is combining aerosol techniques and sol–gel chemistry, which is known as “aerosol-assisted sol–gel” (AASG) (Debecker et al., 2018). Dehydration of enzymes by spray-drying, however, has a downside: it can cause thermal denaturation leading to the loss of enzymatic activity (Alloue et al., 2007). The denaturation of protein during this process can be minimized by adding additives, such as polysaccharide, proteins and salts. These additives, also called wall material, can be a physical barrier to protect enzymes against the heat during the spray-drying process (Tonon, Grosso & Hubinger, 2011; Carneiro et al., 2013; Estevinho et al., 2013). Therefore, apart from being the support material for enzyme immobilization, sago can also act as a wall material during the spray-drying process. In order to verify the effectiveness of the enzyme dehydration process, the activity of the enzyme that has been stored at various temperatures should be monitored.

The purpose of this research is to enhance the stability of ARM lipase using sago as the immobilization carrier together with the spray-drying method. ARM lipase was isolated from *Geobacillus* sp. strain ARM. This thermophilic lipolytic bacterium strain was isolated from cooking oil contaminated soil. ARM lipase exists in two chains and the crystal structure is available at PDB (4FMP). This lipase is thermostable, organic solvent tolerant and prefers medium and long-chain fatty acids as a substrate (Ebrahimpour et al., 2011; Rahman et al., 2015). Lipase or triacylglycerol acylhydrolase (EC 3.1.1.3) is an enzyme that catalyzes the hydrolysis of long chain triacylglycerides forming diacylglycerides, monoacylglycerides, glycerols and free fatty acids. The reaction of lipase is reversible in water-limiting condition. Therefore, lipase can catalyze two other reactions, that is, synthesis of ester and interesterification (Zaidan et al., 2010; Ebrahimpour et al., 2011). Some lipases may have regiospecificity (selective to ester bonds in position *sn*-1, 3 of a triacylglycerol), stereospecificity (selective to *sn*-1 or *sn*-2 position of a triacylglycerol) and substrate specificity (length of fatty acid) while some others do not (non-specific lipase). These special features of lipase can be exploited to alter the existing lipids and produce novel structured lipids and ester products for applications in the cosmetics, nutritional, food and pharmaceutical industries (Akoh & Min, 2002; Rahman et al., 2015).

## Materials and Methods

### Materials

Chemicals and solvents used were analytical grade, purchased from Merck. Ampicillin and IPTG were purchased from Sigma-Aldrich (St. Louis, MO, USA). Olive oil was purchased from Bertolli (Lucca, Tuscany, Italy). Food grade sago and sago powder were obtained from a local bakery (Malaysia).

### Bacterial source

The recombinant plasmid used in this study was obtained from Ebrahimpour et al. (2011). The mature ARM lipase gene was amplified and cloned into pTrcHis2 TOPO expression system (Invitrogen, Carlsbad, CA, USA) and transformed into *E. coli* TOP10 host cells (Ebrahimpour et al., 2011).

### Expression of recombinant ARM lipase

The expression was done in 1 L LB broth containing 50 µg/mL ampicillin in 3 L conical flask. The culture was incubated at 37 °C with 250 rpm shaking in INFORS HP (Ecotron, Wixom, MI) incubator shaker. The expression was induced with 1 mM IPTG when optical density A_600_ reached 0.5. The culture was harvested after 20 h of cultivation.

### Enzyme purification

ARM lipase expressed in TOP10 *E. coli* was purified by affinity chromatography technique using Äkta Explorer (GE Healthcare, Uppsala, Sweden). The harvested cell pellet was resuspended in binding buffer (20 mM sodium phosphate, 0.5 m NaCl, 20 mM imidazole, pH 7.4). The cell suspension was sonicated using a Branson Digital Sonifier (6 min with 30 s lapse; amplitude: 30%) and cleared by centrifugation (12,000×  g, 30 min, 4 °C). The crude protein was loaded into 2 tandem 5 mL HisTrap HP column (GE Healthcare) at a flow rate of 1 mL/min. The column was then washed with 5 column volumes of binding buffer. Finally, the bound enzyme was eluted with elution buffer (20 mM sodium phosphate, 0.5 M NaCl, 0.5 M imidazole, pH 7.4) by a linear gradient. The protein content was determined by Quick Start™ Bradford protein assay (Biorad, Hercules, CA) and tested for lipase activity.

### Lipase assay

The lipase activity was assayed by a colorimetry method (Kwon & Rhee, 1986). Lipase (10 µL of free enzyme or 10 mg of immobilized enzyme), 2.5 mL of olive oil emulsion and 20 µL of 20 mM CaCl_2_ was incubated in a water bath shaker at 70 °C with an agitation rate of 200 rpm, for 30 min. The enzyme reaction was stopped by adding HCl (1 mL). Free fatty acid was extracted by adding isooctane (5 mL) and detected by adding copper-pyridine into the reaction mixture (1 mL). Absorbance was read at 715 nm wavelength. One unit of lipase activity was defined as the rate of 1 µmole fatty acid released per minute.

### Immobilization of ARM lipase in sago

1 g of sago powder (1% w/v in distilled water) was heated at 80 °C for 30 min. The solution was cooled to room temperature. Purified ARM lipase (1 mg) was then added. The mixture was stirred (200 rpm) at room temperature for 30 min. The mixture was then spray-dried using BÜCHI Mini Spray Dryer B-290. The inlet temperature of the spray-dryer was tested at 60 to 150 °C, while the aspirator and pump remain constant, 35 m^3^/h and 2 mL/min respectively. The activity of the immobilized enzyme was then assayed. The immobilization was also tested with 2% sago powder. Lipase spray-dried with the inlet temperature of 80 °C was taken for further characterization.

### Characterization of immobilized lipase

#### Protein staining-light microscope

Samples were stained with Coomassie Brilliant Blue R-250 solution for 5 min and washed using the de-staining buffer (10% methanol and 10% acetic acid) for 24 h. Samples were fixed on glass slides and viewed under a light microscope.

#### Scanning electron microscopy

The morphology of the samples was analyzed using scanning electron microscopy (SEM). The analysis was carried out using JEOL JSM-6400 microscope. Sample particles were coated with gold before analyzed under SEM.

#### Surface area and porosity analysis

Lipase spray-dried with the inlet temperature of 80 °C was taken for analysis. Sago flour and spray-dried gelatinized sago (without lipase) were tested as the control. The analysis was done using Autosorb-1 (Quantachrome) and the software used was AS1Win version 2.01. The surface area was calculated using the Brunauer–Emmett–Teller (BET) method. The pore volume and pore size were calculated using the Berrett-Joyner-Halenda (BJH) method.

#### Effect of temperature on enzyme activity

Lipase assay was performed for the immobilized ARM lipase at 40 °C to 90 °C with 10 °C intervals.

#### Effect of temperature on enzyme stability

The immobilized lipase samples were incubated at 60 °C, 70 °C and 80 °C for 1–5 h prior to lipase assay. The residual enzyme activity was assayed as described previously. For storage stability, the immobilized enzyme was stored at −20 °C, 10 °C and room temperature, and the residual enzyme activity was assayed every month.

#### Effect of pH on enzyme activity

The effect of pH on immobilized ARM lipase activity was studied at pH 5 to pH 10. Lipase activity was assayed as in section 2.5 using buffers with different pH levels; 50 mM sodium citrate buffer for pH 5–6, 50 mM potassium phosphate buffer for pH 6–8, 50 mM Tris-HCl buffer for pH 8–9 and 50 mM glycine-NaOH for pH 9–10.

## Results

### Pure ARM lipase production

ARM lipase was expressed intracellularly in *Escherichia coli* expression system. The expression level was determined by the lipase assay. The recombinant ARM lipase expressed by pTrcHis2 TOPO expression vector has His-tag fused to the enzyme, which has a strong affinity for divalent cations, so that the fusion protein can be purified using metal chelating resins like nickel sepharose (Mohtar et al., 2016). The enzyme recovery obtained was 81%, while the specific activity of the crude enzyme was 100 U/mg and increased by 7-fold to 650 U/mg after purification (Table 1).

**Table 1:** Purification of ARM lipase using affinity chromatography.

Fraction	Total protein (mg)	Total activity (U)	Specific activity (U/mg)	Fold	Yield (%)
Cell lysate	1,058.58	105,514	100	1	100
Purified enzyme	131.76	85,624	650	7	81

### Immobilization of ARM lipase in sago

In this study, the ARM lipase was entrapped into gelatinized sago and spray-dried. The effect of the different inlet temperature of the spray-dryer was studied to determine the inlet temperature that is less damaging for the enzyme. When the inlet temperature was adjusted, the outlet temperature will automatically be changed accordingly. The results in Table 2 shows that when ARM lipase was immobilized in 1% gelatinized sago, the best inlet temperature for spray-drying the immobilized enzyme was at 80 °C to 100 °C (86.3–80.2 U/g). Lipase spray-dried with an inlet temperature of 80  °C was taken for further characterization. Enzyme activity dropped by 42% when the outlet temperature increased from 100 °C to 150 °C. When the sago concentration increased to 2% for the immobilization, the enzyme activity of the immobilized lipase dropped to 68.2 U/g, 69.7 U/g and 58.9 U/g when spray-dried at 60 °C, 80 °C and 100 °C respectively. When the concentration of sago was increased further to 3%, it became too viscous to be spray-dried.

**Table 2:** Enzyme activity of spray-dried immobilized ARM lipase. Values are expressed as mean ± standard error.

**Inlet temperature (°C)**	**Outlet temperature (°C)**	**Enzyme activity (U/g)**
60	40	73.4 ± 3.6
70	45	66.2 ± 3.4
80	55	86.3 ± 6.0
90	70	86.1 ± 5.8
100	75	80.2 ± 3.7
130	83	62.9 ± 5.3
150	100	46.8 ± 2.6

### Characterization of ARM lipase immobilized in gelatinized sago solution

#### Protein staining and light microscope

**Figure 1:**
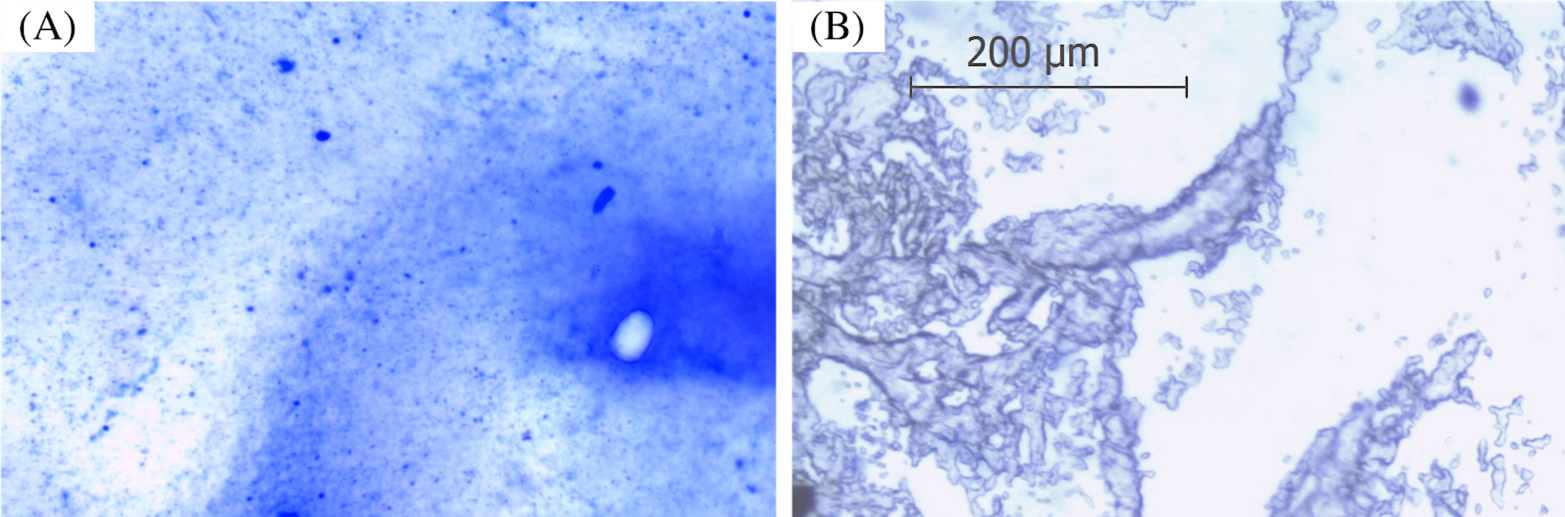
Protein stained with Coomassie Blue viewed under a light microscope with 100x magnification. (A) Spray-dried immobilized ARM lipase. (B) Spray-dried gelatinized sago.

Coomassie Brilliant Blue R-250 was used to visualize the presence of ARM lipase in the gelatinized sago. The dye molecules bind to proteins via ionic interactions between the negatively charged sulfonic groups of the dye and the positive charges of the protein amine groups forming the protein-dye complex (Tal, Silberstein & Nusser, 1985). The sensitivity of Coomassie R-250 is 0.1 µg of protein (Wang, Li & Li, 2007). The result shows the presence of protein, ARM lipase, entrapped in the gelatinized sago. The tiny blue dots shown in Fig. 1A are the protein-dye complexes, which were not present in the control (Fig. 1B).

#### Scanning electron microscope

**Figure 2:**
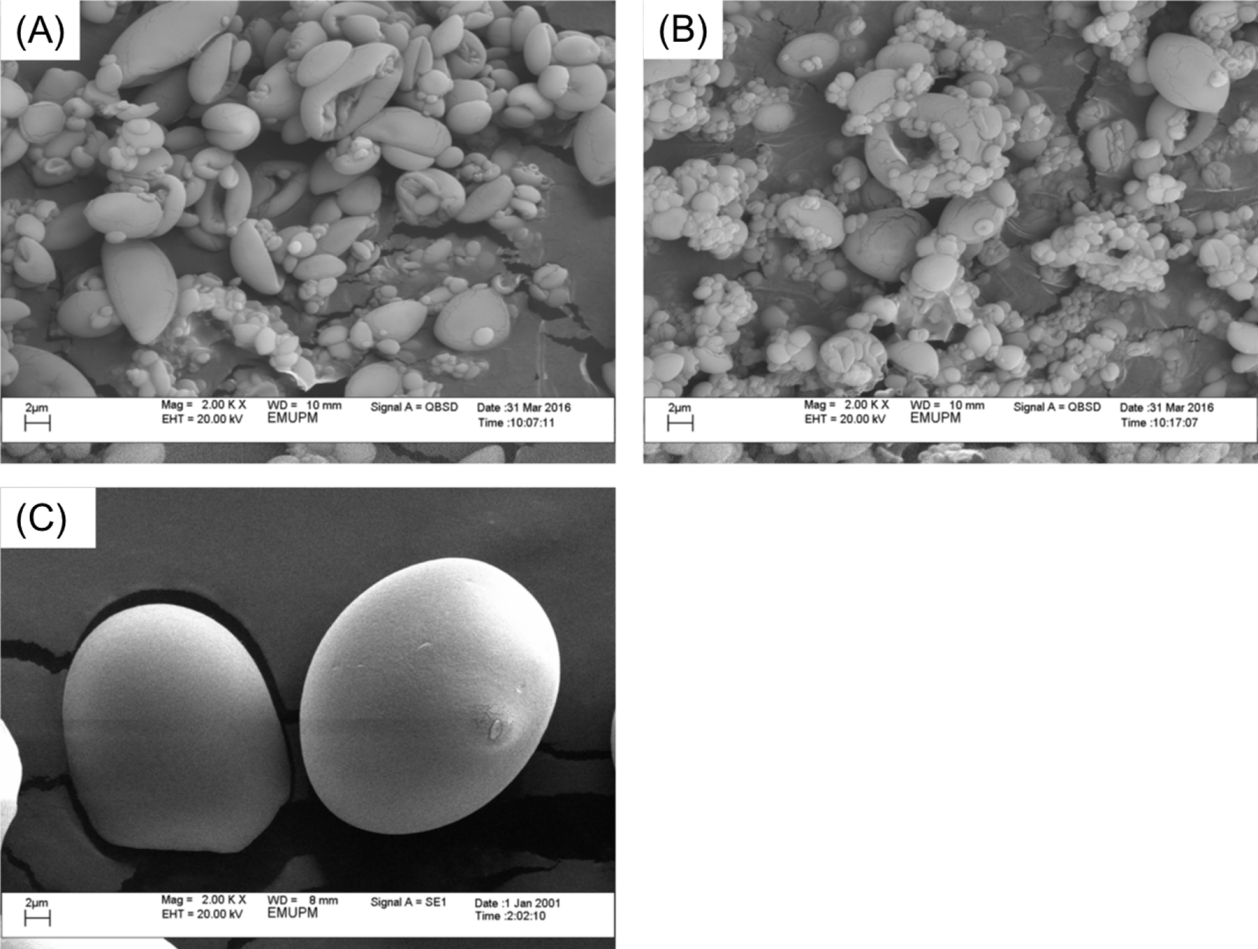
Scanning electron microscope images with 2,000× magnification. (A) Spray-dried gelatinized sago. (B) Spray-dried immobilized lipase on gelatinized sago. (C) Sago flour.

The morphology of sago and immobilized ARM lipase were observed using Scanning Electron Microscope (SEM) (Fig. 2). The spray-dried gelatinized sago (Figs. 2A and 2B) is seen shrunken and irregular smaller sizes (1–6 µm) as compared to untreated sago powder that has the distinct truncated oval sago granules morphology (17–30 µm) (Fig. 2C). The texture of the spray-dried products particles was finer compared to the untreated sago powder. During gelatinization, sago granules would absorb water and swell. When spray-dried afterword, the starch became dehydrated. That is why the structure shrunk and shriveled. However, from the SEM results, the presence of the enzyme entrapped could not be observed due to the very low amount of enzyme and also the size of the protein (Fig. 2A).

#### Surface area and porosity analysis

The surface area of the samples was calculated using the BET method and the pore volume and pore size were calculated using BJH method (Table 3). The surface area and pore volume of the sago powder increased drastically after gelatinization and spray-drying, although the pore radius slightly decreased. After the addition of lipase, the surface area, pore volume and pore radius were reduced.

**Table 3:** BET surface area and BJH adsorption summary.

**Sample**	**Surface area**^a^**(m**^2^**/g)**	**Pore volume**^b^**(cm**^3^**/g)**	**Pore radius**^b^**(Å)**
Sago flour	0.48	0.00	21.80
Spray-dried gelatinized sago^c^	5.42	0.02	17.24
Immobilized lipase^c^	1.79	0.01	15.45

**Notes.**

a BET, (Brunauer, Emmett and Teller) method.

b BJH, (Barrett, Joyner and Halenda) method.

c Spray-dry inlet temperature: 80 °C.

#### Effect of temperature on enzyme activity

**Figure 3:**
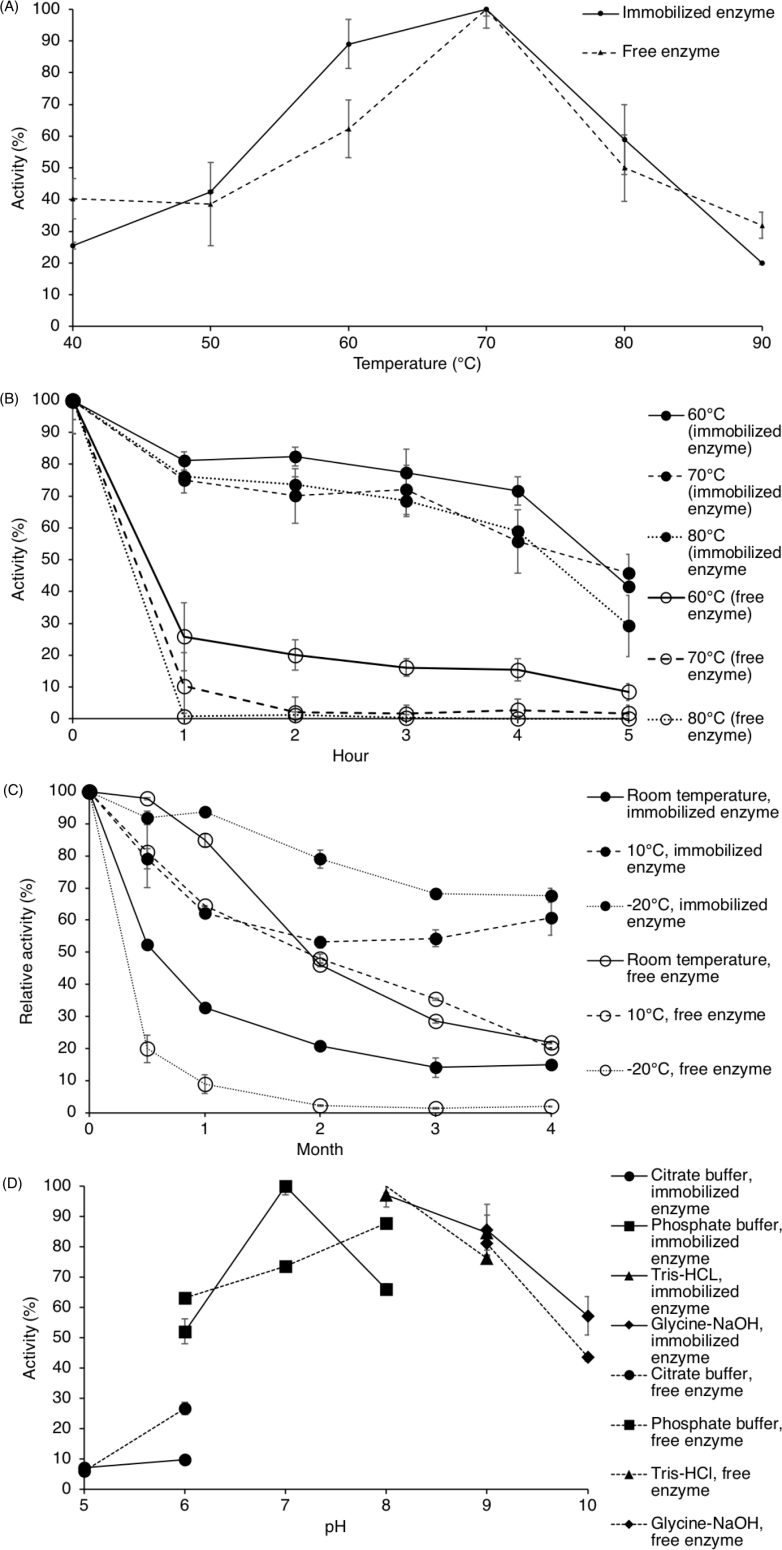
Lipase activity of immobilized enzyme and free enzyme. (A) Effect of temperature on enzyme activity. (B) Effect of temperature on enzyme stability. (C) Enzyme stability for storing at room temperature, 10 °C and −20 °C. (D) Effect of pH on enzyme activity. Note: Data represents mean ± SE (*n* = 3).

As seen in Fig. 3A, the effect of temperature on the activity of the free enzyme and immobilized ARM lipase was comparable. The immobilized enzyme shows increased activity and the maximum can be observed at 70 °C (102.67 U/g for the immobilized enzyme and 234.22 U/ml for the free enzyme). But as the temperature increased further, lipase activity decreased drastically. The residual enzyme activity dropped to 45% when assayed at 80 °C and 21% at 90 °C.

#### Effect of temperature on enzyme stability

To test thermal stability, the enzyme was heated from 60 °C to 80 °C, and enzyme activity was assayed every hour for 5 h. Referring to Fig. 3B, the immobilized enzyme activity was slowly dropping throughout the 5 h when treated at those temperatures. The half-life of the immobilized enzyme at 60 °C was 4.5 h while at 70 °C and 80 °C, 4 h. The activity of the free enzyme depleted drastically, even from the first hour.

During storage of immobilized ARM lipase, the activity for the immobilized enzyme dropped gradually when kept at 10 °C and at −20 °C within the first 4 months while the activity for the free enzyme reduced drastically (Fig. 3C). The half-life of the immobilized enzyme at 10 °C and room temperature was 9 months and 2 weeks, respectively.

#### Effect of pH on enzyme activity

Lipase assay was done at pH 5 to pH 10 using four types of buffers in order to determine the pH preference of ARM lipase. Both free enzyme and immobilized lipase were tested for comparison (Fig. 3D). The highest activity was observed at pH 8 in Tris-HCl buffer for the free enzyme (179.0 U/ml) while for immobilized lipase at pH 7 in phosphate buffer (103.37 U/g). Both free enzyme and immobilized lipase can retain at least 50% of activity at pH 6 to pH 10.

## Discussions

### Immobilization of ARM lipase in sago

Lipase has been used in many food-related applications, such as to produce a cocoa butter substitute in confectionary products and a human milk fat substitute in infant formula (Akoh & Min, 2002). Thus, the use of food grade material like sago as the immobilization support for lipase would be very suitable for the utilization of the enzyme in food industry. Moreover, spray-drying can be damaging to heat labile substances like enzymes. Therefore, enzyme entrapment in sago provides protection during the spray-drying process. However, the increase in sago concentration might cause the active site of the enzyme to be hindered, or limit the mobility of the enzyme and substrate, thus reduced the activity of the immobilized ARM lipase (Trusek-Holownia & Noworyta, 2015). The drop in immobilized enzyme activity when the outlet temperature was increased is probably due to the temperature preference of ARM lipase, as the optimum temperature for enzyme activity is at 65 °C and this enzyme is only stable up to 80 °C (Ebrahimpour et al., 2011). Purified enzymes were used in these experiments to ensure the accuracy and validity of the results, because the contaminants might affect the enzyme activity and stability.

#### Characterization of ARM lipase immobilized in gelatinized sago solution

The increment in the surface area and pore volume of spray-dried gelatinized sago make it suitable for enzyme entrapment. The immobilization of lipase in the sago was indicated by decreases in the surface area, pore volume and pore radius (Zdarta et al., 2015). This shows that the lipase has occupied the surface and pores of the gelatinized sago. This result is supported by the Coomassie Brilliant Blue R-250 staining of the protein where blue specks of protein-dye complex were seen scattered in the gelatinized sago sample and also the lipase activity assay as the evidence of enzyme immobilization. Compared to synthetic compound, the surface area of spray-dried gelatinized sago was way smaller (5.241 m^2^/g). The surface area and pore volume of synthetic immobilization support like double layered hydroxide (Mg/Al-NO_3_^−^), modified mica and Eupergit C are larger (28–60 m^2^/g and 0.023–0.128 cm^3^/g), and therefore would load more enzyme in the structure (Abdul Rahman et al., 2004; Abdul Rahman et al., 2008; Othman et al., 2008; Çiftçi, Fadiloǧlu & Göǧüş, 2009; Lam, Lee & Mohamed, 2010; Zaidan et al., 2010). The surface area and pore size are important to immobilized the enzyme on those synthetic materials via the adsorption method (Schlipf, Rankin & Knutson, 2013). However, in this research, the enzyme was entrapped in the gelatinized sago structure; therefore, the surface area and pore size of the support material might not be as critical.

The activation energy of the lipase was achieved as the assay temperature rose approaching 70 °C and therefore, the rates of reaction catalyzed by the enzyme increase (Voet & Voet, 2011). However, further increment of the temperature decreased the enzyme activity. This may have caused by protein denaturation. The elevated temperature may break some of the weak bonds. The structure of the enzyme, including its active site, would be disrupted and therefore the denatured enzyme is not functional, causing a loss in enzyme activity (Bischof & He, 2005; Bisswanger, 2014). The half-life of the immobilized enzyme in this study was better when compared to the free ARM lipase. The half-lives of the free ARM lipase were less than an hour at 60 °C, 70  °C and 80 °C, which was similar to values reported by Ebrahimpour et al. (2011) (Fig. 3B). The entrapment of lipase in sago protected the enzyme from heat denaturation, thus making the immobilized enzyme more stable than the free enzyme at high temperature. This would mean that the immobilized enzyme would be good for applications that require a longer reaction time at high temperature, for example, interesterification and acidolysis for structured lipid production, as it may take 6 h to completely deprived the enzyme activity at 80 °C (Gunstone, 2001; Kim & Akoh, 2015).

One of the main purposes of enzyme immobilization is to extend the shelf-life compared to the free enzyme. This feature would be tremendously beneficial for industrial applications. This study has shown that the entrapment of lipase in sago has prolonged the shelf-life of the enzyme compared to the free enzyme when stored at −20 °C and 10 °C. According to Costa-Silva et al. (2016), the effects of stabilization may be due to the interaction between the enzymes and the supporting material although the mechanism of stabilization in the solid state are still not completely understood. As reported by Belghith, Ellouz Chaabouni & Gargouri (2001), the humidity of the spray-dried enzyme was higher when samples were stored at higher temperature (30 °C) and studies have shown that lipase activity dropped when the spray-dried enzyme was kept in an atmosphere with higher water activity (Belghith, Ellouz Chaabouni & Gargouri, 2001; Alloue et al., 2007). Alloue et al. (2007) stated that the presence of water might disrupt the enzyme conformation during storage. Studies have shown that enzymes that were spray-dried at higher temperatures (118–200 °C) and kept at controlled humidity were stable for 8 to 18 months at 4 °C (Belghith, Ellouz Chaabouni & Gargouri, 2001; Alloue et al., 2007). Other than storing at low temperature, it is possible to extend the half-life of the samples if kept in a controlled humidity environment. However, when the free enzyme was kept at −20  °C, the activity dropped significantly when assayed. This was caused by the drastic temperature change during the freeze and thaw process that damaged the protein and reduce its activity (Pikal-Cleland et al., 2000). This effect was not observed in the immobilized lipase as the addition of sago seems to protect the enzyme against sudden temperature changes.

In general, enzymes are sensitive to pH changes. The pH of the enzyme environment would affect substrate binding, catalytic reaction, ionization of substrate and the protein structure, which subsequently would influence the enzyme activity (Voet & Voet, 2011). It is expected for the immobilized lipase to be active in a broad range of pHs as the free enzyme. In addition, the polymer (gelatinized sago) acted as a membrane to the entrapped ARM lipase. Leading to temperature, pH and organic solvent stability (Minteer, 2011). A broad pH range would be an excellent feature for the enzyme across industries. For example, applications in food processing may require the reaction to be performed under acidic conditions, while detergent productions may require the reaction to be performed under alkaline conditions.

## Conclusions

The study has shown that the entrapment of ARM lipase in gelatinized sago efficiently preserved the enzyme for storage and stabilized the enzyme at high temperature (60 °C to 80 °C) based on its catalytic activity in comparison to the free enzyme. The immobilized enzyme maintains its optimum temperature for enzyme activity at 70 °C and a pH preference from pH 7 to pH 9. The spray-drying process has been shown to be an effective method to dehydrate the enzyme while keeping good enzymatic activity under storage at 10  °C and at −20 °C, which will be very useful for industrial or scientific use. Overall, the thermal and pH characterization results have shown that immobilized lipase in support material which is safe for consumption and biodegradable such as sago is very promising in food manufacturing applications and many more.

##  Supplemental Information

10.7717/peerj.6880/supp-1 Dataset S1 Raw data for Table 3

10.7717/peerj.6880/supp-2 Dataset S2 Raw data for Fig. 3

## Additional Information and Declarations

### Data Availability

The following information was supplied regarding data availability: Raw data are available in the Supplemental Files.
